# Anti-Biofilm Effects of Rationally Designed Peptides against Planktonic Cells and Pre-Formed Biofilm of *Pseudomonas aeruginosa*

**DOI:** 10.3390/antibiotics12020349

**Published:** 2023-02-08

**Authors:** Young-Min Kim, Hyosuk Son, Seong-Cheol Park, Jong-Kook Lee, Mi-Kyeong Jang, Jung Ro Lee

**Affiliations:** 1 Department of Chemical Engineering, Sunchon National University, Suncheon 57922, Republic of Korea; 2 Department of Exhibition and Education, National Marine Biodiversity Institute of Korea, Seocheon 33662, Republic of Korea; 3 LMO Team, National Institute of Ecology (NIE), Seocheon 33657, Republic of Korea

**Keywords:** *Pseudomonas aeruginosa*, planktonic cells, antimicrobial peptide, antimicrobial resistance, biofilm

## Abstract

Biofilms are resistant to antibiotics and are a major source of persistent and recurring infections by clinically important pathogens. Drugs used for biofilm-associated infections are limited because biofilm-embedded or biofilm-matrix bacteria are difficult to kill or eradiate. Therefore, many researchers are developing new and effective antibiofilm agents. Among them, antimicrobial peptides have an attractive interest in the development of antibiofilm agents. The present study evaluated the effects of 10 synthetic peptides on growth inhibition, inhibition of biofilm formation, and biofilm elimination in drug-resistant *Pseudomonas aeruginosa*. The planktonic cell growth and biofilm formation were dose-dependently inhibited by most of the peptides. WIK-14 eliminated preformed biofilm masses by removing carbohydrates, extracellular nucleic acids, proteins, and lipids constituting extracellular polymeric substances. The results demonstrated that WIK-14 and WIKE-14 peptides might provide novel therapeutic drugs to overcome multidrug resistance in biofilm-associated infections.

## 1. Introduction

Most microbes can evolve several survival strategies to resist antimicrobial treatments, adapt to extreme environmental stresses, and survive the host’s immune responses [1,2,3]. A biofilm is a community of microorganisms attached to biotic (living organisms) or abiotic (non-living substances) surfaces and is surrounded by a self-produced extracellular polymeric substance (EPS) [4,5,6,7]. Biofilm is one of many global healthcare concerns; it causes nearly 80% of nosocomial infections. Microbes adhere and form a biofilm on different host tissues such as epidermal cells, mucosal surfaces, and teeth and on medical devices such as bone joints, breast implants, cardiovascular valves, contact lenses, endotracheal tubes, urinary catheters, orthodontal prosthetics, and pacemakers [8,9,10,11].

Biofilm formation is divided into four steps: the adhesion stage, wherein planktonic cells attach to a surface; the sessile growth stage, wherein the cells form microcolonies; the secretion stage of EPS, which includes exogenous DNA, lipids, polysaccharides, and proteins; the maturation stage, where a tower structure is formed [12,13]. These cyclical stages are repeated by the dispersion or disassembly of the biofilm via disruptive factors, including nucleases, phenol-soluble modulins, proteases, and regulators [14]. In this disrupting process, biofilms can shed fragments and individual cells into the bloodstream and surrounding tissues, causing many acute and chronic infections [15,16].

Antimicrobial peptides (AMPs) have been considered attractive antibiofilm agents due to their broad spectrum of antimicrobial actions, anti-inflammatory activity, and synergistic effects with few antibiotics [17,18,19,20]. AMPs are biomolecules that are composed of 12–50 amino acids. They are well-known to have potent antibacterial, antifungal, antiviral, and antitumor activities and avoid resistance due to their membranolytic actions [21,22]. Moreover, some AMPs have potent antibiofilm activity against multidrug resistant bacteria, either by preventing with biofilm formation via interfering with the adhesion of bacteria to the surface or destroying mature biofilms by detaching the EPS or killing the bacteria present [23,24,25].

The present study was performed to assess the antibiofilm properties of ten previously reported AMPs [26] against drug-sensitive and drug-resistant *P. aeruginosa* strains using in vitro phenotypic and EPS analyses.

## 2. Results and Discussion

### 2.1. Antibacterial Activity of Peptides against Clinical Isolates of P. aeruginosa

To confirm the clinical applicability of the designed peptides, the MIC values were determined for five drug-resistant *P. aeruginosa* strains isolated from patients with otitis media. As shown in Table 1, antibiotics containing ciprofloxacin, ceftazidime, and tobramycin had minimum inhibitory concentrations (MICs) ranging from 0.25 to 1 µM against drug-sensitive *P. aeruginosa* (ATCC 27853). Despite this, the majority of their MICs against drug-resistant *P. aeruginosa* (DRPa) 3904, 4830, 4891, 4007, and 5018 strains were significantly increased above 256 µM, indicating that five DRPa strains are multidrug resistant *P. aeruginosa* because ciprofloxacin, ceftazidime, and tobramycin belong to different classes of antibiotics, e.g., fluoroquinolone, beta-lactam, and aminoglycoside, respectively. Since the modes of action of the three antibiotics differ, the emergence of a bacteria resistant to all of them represents a serious risk of bacterial infections. Ten peptides used in this study exerted a potent antibacterial activity with MICs ranging from 2 µM to 16 µM in drug-sensitive and drug-resistant *P. aeruginosa* strains. Their MICs for drug-resistant strains were equal to or lower than those of drug-sensitive strains (Table 1). In addition, the antibacterial activity of the 14-mer peptides was better than that of the 10-mer peptides, and the MICs of peptides with Trp located at the amino terminus (WIK-10, WIR-10, and WIK-14) were lower than that of the others. Thereby, the 10 designed peptides are effective against the clinically isolated DRPa. Among them, KIW-14, KWI-14, and WIK-14 peptides are expected to be developed as new antibiotics to treat drug-resistant *P. aeruginosa* infections. Moreover, our previous report has shown that these peptides are non-toxic at 100 µM [26].

### 2.2. Growth Inhibition of Peptides in Various Ionic Strength

**Figure 1:**
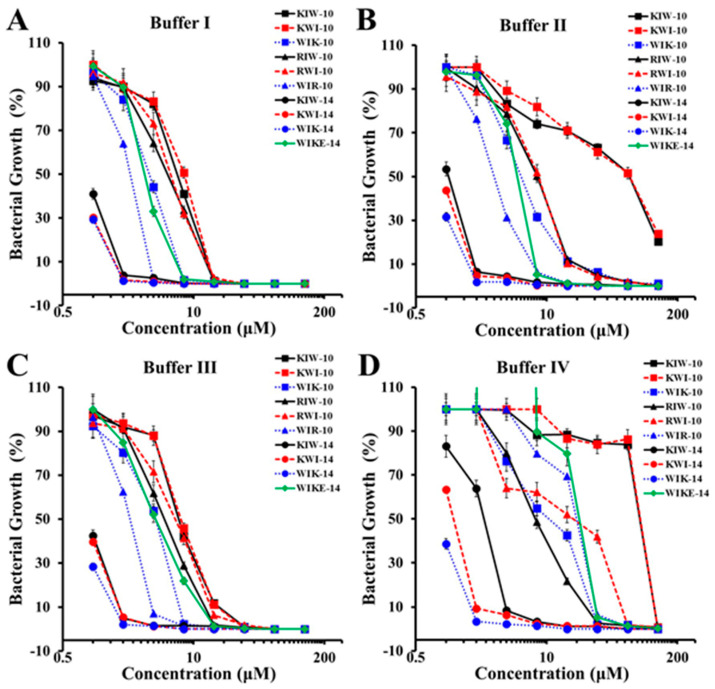
Antibacterial activity of peptides in different ionic buffers against DRPa 4007. (**A**) Buffer I: 10 mM HEPES, 10 mM NaCl, pH 7.4 + 10% MH; (**B**) buffer II: 10 mM HEPES, 150 mM NaCl, pH 7.4 + 10% MH; (**C**) buffer III: 10 mM HEPES, 5 mM MgCl_2_, pH 7.4 + 10% MH; (**D**) buffer IV: 10 mM HEPES, 150 mM NaCl, 5 mM MgCl_2_, pH 7.4 + 10% MH.

The antimicrobial activity of AMPs must be sustained in physiological environments to be administered to the human body. In particular, Ca^2+^ and Mg^2+^ ions are essential to regulate metabolism and to maintain homeostasis. The activity of several antimicrobial peptides disappeared or decreased in the presence of high monovalent salt and divalent cations [26,27,28]. The antibacterial activities of the synthesized peptide variants were further compared in various buffer conditions. The ionic strength of the peptides was determined in the presence of 10 and 150 mM NaCl with and without 5 mM MgCl_2_. As shown in Figure 1, all peptides in the buffer inhibited the growth of tested bacteria by more than 95%, even at a concentration of less than 32 µM. Hydrophilic peptides with ten lysine-containing amino acids (Lys, K), such as KIW-10, KWI-10, and WIK-10, inhibited antibacterial activity by more than 50% in buffers I, II, and IV when compared with buffer I. Furthermore, hydrophilic peptides with ten arginine-containing amino acids (Arg, R), such as RIW-10, RWI-10, and WIR-10, inhibited antibacterial activity by 20~50% when compared with the activity in buffer I. In addition, the activities of peptides with a 14-mer length, such as KIW-14, KWI-14, WIK-14, and WIKE-14, were nearly identical to those in buffer I. These findings suggested that KIW-10, KWI-10, WIK-10, RIW-10, RWI-10, and WIR-10 could be used for external preparation, unrelated to body fluids. Due to their ability to function under salt conditions in vivo, KIW-14, KWI-14, WIK-14, and WIKE-14 can be used for both external and internal preparation.

### 2.3. Growth Inhibition of P. aeruginosa Planktonic Cells

**Figure 2:**
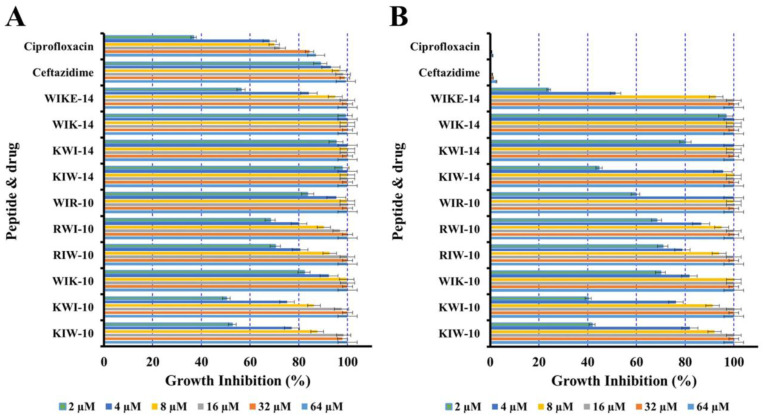
Growth-inhibitory effects of peptides and antibiotics in planktonic *P. aeruginosa* ATCC 27853 (**A**) or DRPa 4007 (**B**).

To investigate the growth inhibition of peptides and antibiotics against planktonic cells, the antimicrobial assay was performed in a full medium of brain heart infusion (BHI) with drug-sensitive *P. aeruginosa* (ATCC 27853) and drug-resistant DRPa-4007 cells. As shown in Figure 2, two conventional antibiotics, ciprofloxacin and ceftazidime, inhibited the growth of drug-sensitive *P. aeruginosa* at very low concentrations, although their antibacterial activity was remarkably inhibited for DRPa-4007. At concentrations greater than 4 µM, all peptides inhibited the growth of both *P. aeruginosa* strains (Figure 2). Although the media of the antimicrobial assay was changed, the results showed the antibacterial activity pattern shown in Table 1. The effective removal of bacterial planktonic cells would significantly prevent bacterial biofilm formation. In particular, drug-resistant bacteria can easily form biofilms because they cannot be killed by conventional drugs, but antibacterial peptides can kill both drug-sensitive and resistant bacteria, so it can be used as a biofilm prevention agent.

### 2.4. Inhibition of Biofilm Formation and Reduction on Preformed Biofilm

**Figure 3:**
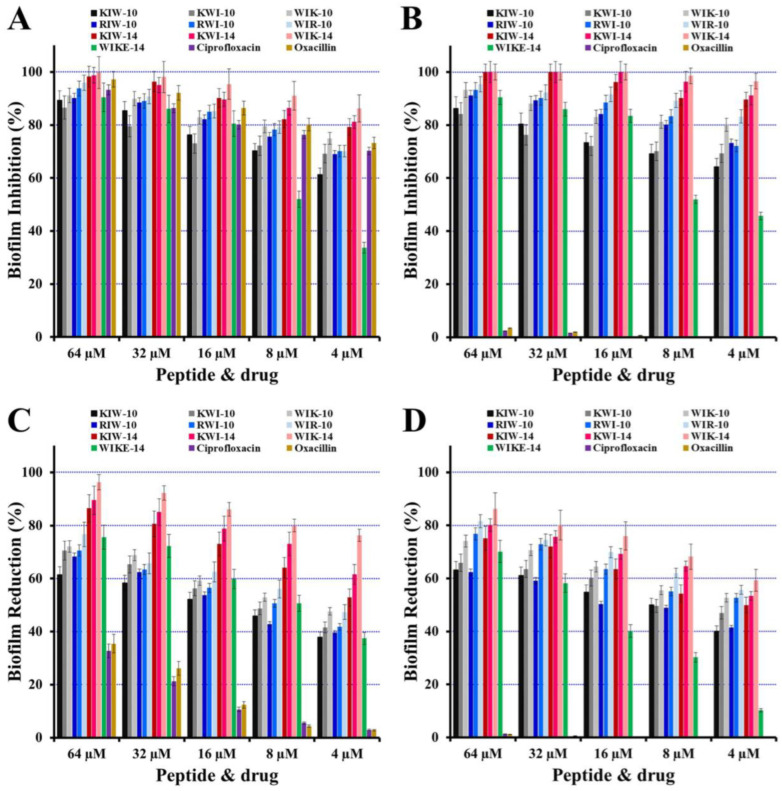
Biofilm-inhibitory (**A**,**B**) and reductive activities (**C**,**D**) of peptides and antibiotics in drug-sensitive *P. aeruginosa* ATCC 27853 (**A**,**C**) or drug-resistant DRPa 4007 (**B**,**D**).

Phenotypic analyses were performed for *P. aeruginosa* (ATCC 27853 and DRPa 4007) biofilm to investigate the effects of peptides in biofilm formation using the crystal violet biomass staining method. Conventional antibiotics, ciprofloxacin and oxacillin, as shown in Figure 3A,B, inhibited biofilm formation by drug-sensitive *P. aeruginosa* ATCC 27853 in a dose-dependent manner. However, at a concentration of 64 µM, the drug-resistant strains (DRPa 4007) only inhibited biofilm formation by 2.3% of ciprofloxacin and 3.4% of oxacillin. In the presence of all peptides, however, two strains were inhibited in a dose-dependent manner.

After 48 h of biofilm formation, different antimicrobial samples (ciprofloxacin, oxacillin, or peptides) were added to evaluate the reductive activity on preformed biofilms, followed by an additional 24 h of incubation. As shown in Figure 3C,D, all peptides significantly contributed to the reduction of the preformed biofilm in both *P. aeruginosa* strains. WIK-14, in particular, was the most effective at removing preformed biofilm, with a reduction of 96.3% and 86.2% in *P. aeruginosa* ATCC 27853 and DRPa 4007, respectively, at 64 µM. In addition, antibacterial activity and the antibiofilm effect of peptides (WIK-10, WIR-10, and WIK-14), in which the Trp residue with an indole aromatic ring is located at the N-terminus, were better than other peptides. Trp residues can interact with the interface region of a membrane and allow to anchor the peptide to the bilayer surface [27]. This effect was observed in many tryptophan (Trp)-containing AMPs. Therefore, we propose that the first Trp residue exhibits excellent antimicrobial and antibiofilm activity by interacting simultaneously with the head groups and the acyl chains of the lipid.

### 2.5. Effects of Peptides on Biofilm Components

**Figure 4:**
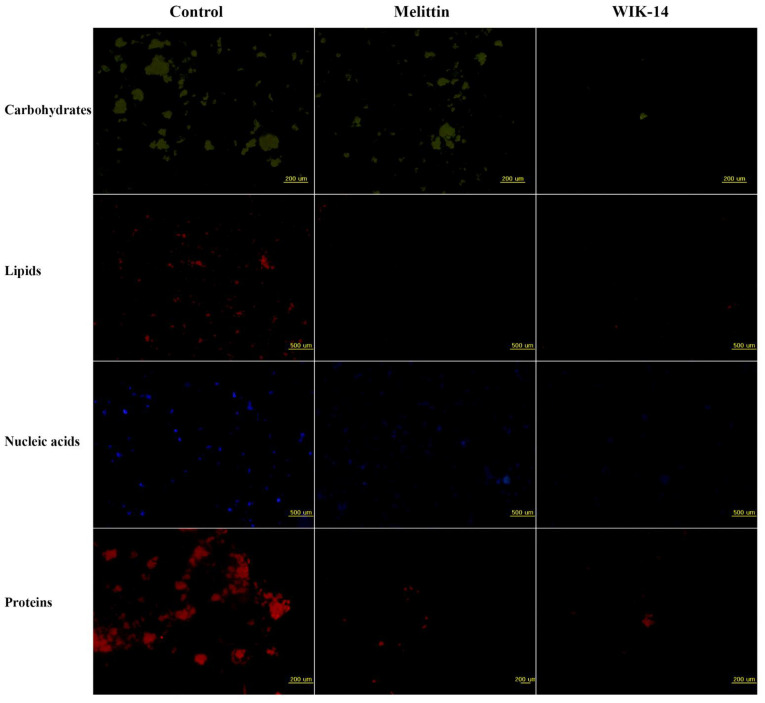
Eliminative action of peptides in biofilm extracellular polymeric substances (EPS). Biofilm EPS of *P. aeruginosa* DRPa 4007 in the absence or presence of peptides (64 µM) was stained by FITC-ConA for carbohydrates, Nile red for lipids, DAPI for nucleic acids, and SYPRO red for proteins and observed under fluorescence microscopy.

To investigate how peptides reduce bacterial biofilms, fluorescent dyes were applied after 24 h incubation of melittin and WIK-14, followed by visualization using fluorescence microscopy (Figure 4). The dyes that were used included those that can bind to carbohydrates (fluorescein isothiocyanate-concanavalin A, FITC-ConA), lipids (Nile red), DNA (4′,6-Diamidine-2′-phenylindole dihydrochloride, DAPI), and proteins (SYPRO red). Melittin did not remove carbohydrates among EPS components, but WIK-14 significantly reduced all components. Results indicated that WIK-14 reduces biofilm biomass by breaking down the network structures of carbohydrates, lipids, and extracellular DNA, owing to their amphipathic structure and cationicity.

### 2.6. Ex Vivo Biofilm Eliminative Effects of Peptides on a Plastic Coverslip

**Figure 5:**
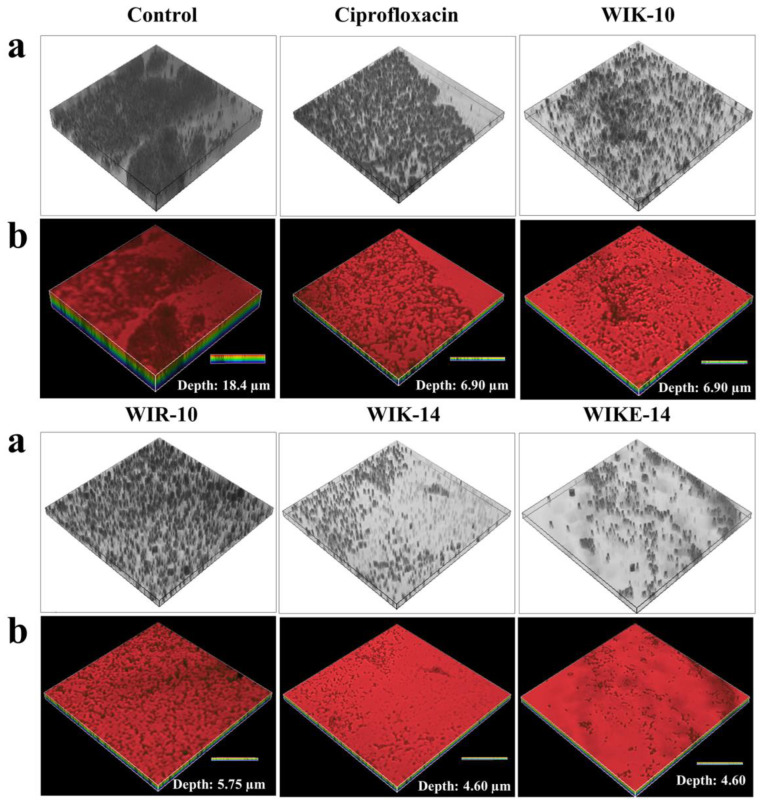
Reductive effects of ciprofloxacin and peptides on biofilm formed by *P. aeruginosa* DRPa 4007 strain using confocal laser scanning microscopy (CLSM). The biofilm reduction in the absence (control) or presence of ciprofloxacin (256 µM) and peptides (64 µM). Biofilm was stained using FilmTracer SYPRO Ruby biofilm matrix stain and observed in bright (**a**) and fluorescent (**b**) fields under CLSM.

To investigate whether ciprofloxacin and peptides can remove preformed biofilm on a plastic coverslip inoculated with *P. aeruginosa* DRPa 4007 cells, FilmTracer SYPRO Ruby biofilm matrix stain solution was used to stain matrices of biofilms. As shown in Figure 5, the treatment with WIK-14 (64 μM) and WIKE-14 (64 μM) resulted in greater fluorescence reduction than the treatment with ciprofloxacin (256 μM). WIK-14 and WIKE-14 peptides eliminated the biofilm depth of 13.8 nm compared with the control in the confocal laser scanning microscopy (CLSM) three-dimensional analysis (Figure 5). We suggest that the excellent biofilm-removal efficiency of WIK-14 and WIKE-14 is due not only to the direct removal of biofilm EPS via the cationic and hydrophobic characters of the peptide but also to the killing of bacteria inside the EPS via their excellent EPS permeability.

### 2.7. Morphological Analysis under Scanning Electron Microscopy (SEM)

**Figure 6:**
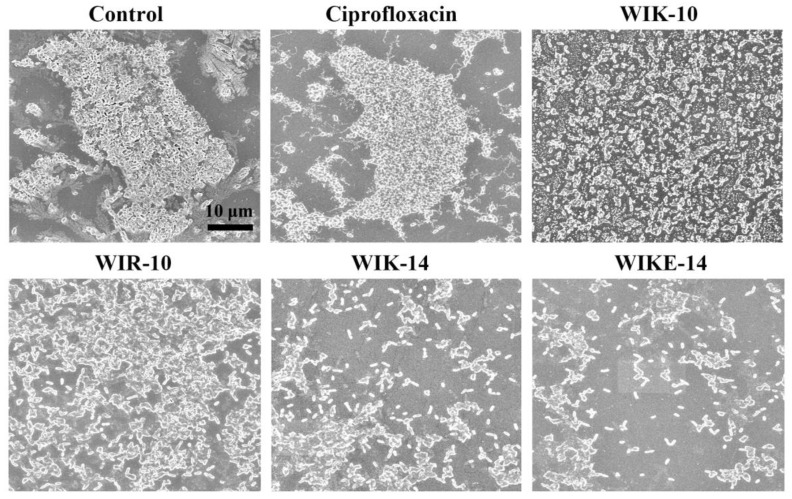
Image analysis of *P. aeruginosa* DRPa 4007 using scanning electron microscopy (SEM). The biofilm reduction on *P. aeruginosa* biofilms in the absence (control) or presence of ciprofloxacin (256 µM) or peptides (64 µM) was observed under SEM.

After the drug-resistant *P. aeruginosa* DRPa 4007 biofilm formed on the plastic coverslips, the biomass of biofilms in the presence of ciprofloxacin and peptides was observed using SEM. The SEM images showed a massive volume of *P. aeruginosa* DRPa 4007 biofilm (Figure 6-control) in non-treated samples as well as a ciprofloxacin-treated coverslip. WIK-10 and WIR-10 treated coverslips showed much bacteria and biofilms. However, the biofilms and bacteria were significantly reduced in the presence of WIK-14 and WIKE-14 peptides. This is consistent with the results of the reductive activity in Figure 3 and the mass measurement in Figure 5.

## 3. Materials and Methods

### 3.1. Materials

Rink Amide Protide resin (0.58 mmol/g), ethyl 2-cyano-2-(hydroxyimino) acetate (Oxyma Pure), and 9-fluorenylmethoxycarbonyl (Fmoc) amino acids were purchased from CEM Co (Matthews, NC, USA). Ciprofloxacin hydrochloride, ceftazidime, oxacillin, tobramycin, SYPRO red, Nile red, FITC-Con A, DAPI, crystal violet, triisopropylsilane, 2,2′-(ethylenedioxy)diethanethiol, trifluoroacetic acid, and N,N′-diisopropylcarbodiimide (DIC) were obtained from Sigma-Aldrich Co. (St. Louis, MO, USA). HPLC-grade water, acetonitrile, and N,N-dimethylformamide (DMF) were bought from Daejung chemicals and metals Co., Ltd. (Siheung-si, Gyeonggi-do, Korea). Other chemicals used were of American Chemical Society reagent grade and used as received without any further purification.

### 3.2. Peptide Synthesis

Sequences of peptides used in the present study are as follows: KIW-10, KKIIKKIWKW; KWI-10, KKIWKKWIKI; WIK-10, WIKKIWKKIK; RIW-10, RRIIRRIWRW; RWI-10, RRIWRRWIRI; WIR-10, WIRRIWRRIR; KIW-14, KKIIKKIIKKIWKW; KWI-14, KKIWKKWIKKIIKI; WIK-14, WIKKIWKKIIKKIK; WIKE-14, WIKKIWKKIIKEIK (Lysine: K, isoleucine: I, tryptophan: W, glutamic acid: E, and arginine: R). Synthesis of all peptides was performed with Fmoc-protected amino acids on an Automated Liberty PRIME Microwave Peptide synthesizer (CEM Co., Matthews, NC, USA). Coupling and deprotection processes were performed with Oxyma/DIC and 20% piperidine (*v*/*v*) in DMF under 90 °C heating. The peptides from the resin were removed by cleavage cocktail solution (trifluoroacetic acid (TFA)/triisopropylsilane/diH_2_O (95:2.5:2.5, *v*/*v*/*v*) for 50 min at 40 °C. The peptide solution was precipitated in ice-cold diethyl ether and the pellet was air-dried. The peptides were resuspended by distilled water and were purified by previously reported methods [26]. The purity of the peptides used in the present study was more than 98%.

### 3.3. Bacterial Strains

*Pseudomonas aeruginosa* ATCC 27853 (drug-sensitive strain) was obtained from the American Type Culture Collection and drug-resistant *P. aeruginosa* (DRPa)-3904, 4830, 4891, 4007, and 5018 were isolated from patients with otitis media in a hospital. DRPa strains were obtained from GC Lab (Yongin-Si, Gyeonggi-do, Korea) for identification and antibiotic susceptibility testing. All strains were grown in a Mueller–Hinton (MH) broth containing 0.5% NaCl under an aerobic condition at 37 °C.

### 3.4. Antibacterial Assay

The MICs of peptides and antibiotics (ciprofloxacin, oxacillin, and tobramycin) were evaluated by a microdilution assay using *P. aeruginosa* ATCC 27853 and drug-resistant (DRPa-3904, 4830, 4891, 4007, and 50418) strains. Pregrown bacteria were suspended in 10 mM sodium phosphate (SP, pH 7.2) supplemented with 20% MH broth. Evaluation of antibacterial effects in variable ionic strengths was performed with the following buffers: 10 mM HEPES, 10 mM NaCl, pH 7.4 + 10% MH (buffer I), 10 mM HEPES, 150 mM NaCl, pH 7.4 + 10% MH (buffer II), 10 mM HEPES, 5 mM MgCl_2_, pH 7.4 + 10% MH (buffer III), and 10 mM HEPES, 150 mM NaCl, 5 mM MgCl_2_, pH 7.4 + 10% MH (buffer IV). Fifty µL of the cell suspension (1 × 10^6^ colony forming unit (CFU)/mL) was added to fifty µL of two-fold serially diluted peptide solutions and antibiotics (0.125–256 µM), followed by incubation for 24 h at 37 °C. The positive growth control was bacterial suspension without peptides and antibiotics and the negative control was only medium. MICs were evaluated by measuring the turbidity of cell solutions at OD_600_ and were defined as the lowest concentration of peptide or antibiotics that inhibited visible bacterial growth. The assays were performed in triplicate.

### 3.5. Growth Inhibition in Planktonic Bacteria

To investigate the growth inhibition of peptides and antibiotics against planktonic *P. aeruginosa* cells, a microdilution assay was applied. Briefly, each peptide or antibiotic (ciprofloxacin and ceftazidime) was two-fold serially diluted in a microtiter 96-well plate (concentration range: 0.125–256 µM) and bacterial suspensions in cation-adjusted brain heart infusion (BHI) broth were subsequently added to each well (1 × 10^7^ CFU/mL). After 24 h incubation at 37 °C with agitation, the growth inhibition was calculated by the absorbance measurement of bacterial turbidity at 600 nm using a Microplate Reader (SpectraMax M5, Molecular Devices, Sunnyvale, CA, USA). The assay was performed in triplicate. The percentage of biofilm inhibition was calculated as follows:

Growth inhibition (%) = 100 − ((OD_600 in sample treatment_ − OD_600 in untreated bacteria_)/(OD_600 in untreated sample_ − OD_600 in untreated bacteria_) × 100)

Sample treatment: adding peptides or antibiotics in bacterial suspensions; untreated bacteria: culture medium without bacteria and samples; untreated sample: bacterial suspension without samples.

### 3.6. Inhibition Assay in Biofilm Formation

The two-fold serially diluted peptides or antibiotics (ciprofloxacin and oxacillin) were incubated with bacterial suspensions in BHI broth supplemented with 2% (*w*/*v*) sucrose (1 × 10^7^ CFU/mL) for 24 h at 37 °C. Sucrose was added to enhance bacterial biofilm formation [28,29]. Unattached bacteria were removed by washing with SP buffer and the remaining biofilm was fixed with methanol for 15 min. After air-drying for 30 min at room temperature, the plate was stained with 0.1% crystal violet (CV) for 5 min, followed by carefully washing with water and adding 95% ethanol to each well. The absorbance of the plate at 570 nm was measured using a Microplate Reader. The experiment was performed in triplicate and the percentage of biofilm inhibition was calculated as follows:

Biofilm inhibition (%) = 100 − ((OD_570 in sample treatment_ − OD_570 in untreated bacteria_)/(OD_570 in untreated sample_ − OD_570 in untreated bacteria_) × 100)

Sample treatment: adding peptides or antibiotics in bacterial suspensions; untreated bacteria: culture medium without bacteria and samples; untreated sample: bacterial suspension without samples.

### 3.7. Reduction Assay on Preformed Biofilm

Bacteria suspended in BHI broth containing 2% (*w*/*v*) sucrose (1 × 10^8^ CFU/mL) were added in a 96-well plate and incubated for 48 h at 37 °C. All wells were rinsed carefully with SP buffer and two-fold serially diluted peptides and antibiotics (ciprofloxacin and oxacillin) were added, followed by exposure for 24 h at 37 °C. After CV staining, the absorbance of the plates was measured at OD_570_, as mentioned above. The assay was performed in triplicate. The percentage of biofilm reduction was calculated as follows:

Biofilm reduction (%) = 100 − ((OD_570 in sample treatment_ − OD_570 in untreated bacteria_)/(OD_570 in untreated sample_ − OD_570 in untreated bacteria_) × 100)

Sample treatment: adding peptides or antibiotics in bacterial biofilm; untreated bacteria: culture medium without bacteria and samples; untreated sample: bacterial biofilm without samples.

### 3.8. Reduction of EPS Components

To investigate the eliminating ability of peptide, EPS components were analyzed using fluorescence microplate and microscopic analyses. After following the processes described in Section 3.7, the biofilm was washed with SP buffer and stained using four fluorescent dyes (DAPI for nucleic acids, Nile red for lipids, FITC-ConA for carbohydrates, and SYPRO red for proteins) [24].

### 3.9. SEM Analysis

DRPa 4007 biofilms were formed on the plastic coverslips (12 mm dimension, SPL life science, Pocheon-si, Korea) for 48 h in BHI broth containing 2% (*w*/*v*) sucrose (1 × 10^8^ CFU/mL) in a 24-well plate. Peptides and ciprofloxacin were incubated at 64 µM and 256 µM, respectively, for 24 h. The coverslips were rinsed using SP buffer and prefixed in 50 mM HEPES buffer (pH 8.0) containing 5% (*v*/*v*) glutaraldehyde for 2 h. After washing with SP buffer, they were dehydrated using the OTTIX shaper (Diapath S.p.A, Bergamo, Italy) and chemical-dried using hexamethyldisilazane. The coverslips coated with gold were observed under a field emission scanning electron microscopy (JSM-7100F, JEOL Ltd., Tokyo, Japan).

### 3.10. CLSM Analysis

To investigate the biofilm-reductive effects of peptides and ciprofloxacin, the biofilm of DRPa-4009 cells was formed on the plastic coverslips for 48 h. After treatments of peptides (64 µM) and ciprofloxacin (256 µM) for 24 h, the coverslips were washed with SP buffer and stained with FilmTracer SYPRO Ruby biofilm matrix stain (Thermo Fisher Scientific Co., Ltd., Seoul, Korea) according to the manufacturer’s protocol. Samples were analyzed by CLSM (A1R HD 25, Nikon, Japan) [30,31].

### 3.11. Statistical Analysis

The mean values of at least four independent determinations ± SD (Student *t*-test) were calculated using Excel software.

## 4. Conclusions

In summary, this study showed that 10 peptides, which are rationally designed AMPs, have an inhibiting effect on biofilm formation and biofilm disruptive activity for preformed biofilms in all tested drug-sensitive and drug-resistant *P. aeruginosa* strains. Moreover, they reduced biofilm presence by detaching the EPS components. WIK-14 and WIKE-14 peptides appear to be interesting candidates in the field of biofilm-associated infections for future drug development strategies. In addition, we believe that our research contributes significantly to the literature because we utilized and explored the relationship between synthetic polypeptides and extracellular polymeric substances to combat multidrug resistance in biofilm-associated infections.

## Figures and Tables

**Table 1:** Growth inhibition of peptides against drug-sensitive and drug-resistant *P. aeruginosa* strains.

Name	MIC (µM)					
	Drug-Sensitive *P. aeruginosa* (ATCC 27853)	Drug-Resistant *P. aeruginosa* (DRPa)				
		3904	4830	4891	4007	5018
KIW-10	16	16	16	8	16	16
KWI-10	16	16	8	8	8	8
WIK-10	8	4	8	8	8	8
RIW-10	16	16	16	8	8	8
RWI-10	16	16	4	4	8	8
WIR-10	4	4	4	4	2	4
KIW-14	2	2	2	2	2	2
KWI-14	2	2	2	1	1	2
WIK-14	2	2	2	1	1	1
WIKE-14	8	16	8	8	8	16
Melittin	2	2	4	4	4	4
Ciprofloxacin	1	>256	64	>256	>256	>256
Ceftazidime	1	>256	128	>256	>256	>256
Tobramycin	0.25	>256	>256	>256	>256	>256

## Data Availability

Not applicable.
